# Crossing-Point Estimation in Human–Robot Navigation—Statistical Linearization versus Sigma-Point Transformation

**DOI:** 10.3390/s24113303

**Published:** 2024-05-22

**Authors:** Rainer Palm, Achim J. Lilienthal

**Affiliations:** 1 Center for Applied Autonomous Sensor Systems (AASS), Department of Technology, Örebro University, SE-701 82 Örebro, Sweden; 2 Technical University Munich (TUM), 80333 Munich, Germany; achim.j.lilienthal@tum.de

**Keywords:** human–robot interaction, Gaussian noise, sigma-point transformation, unscented Kalman filter

## Abstract

Interactions between mobile robots and human operators in common areas require a high level of safety, especially in terms of trajectory planning, obstacle avoidance and mutual cooperation. In this connection, the crossings of planned trajectories and their uncertainty based on model fluctuations, system noise and sensor noise play an outstanding role. This paper discusses the calculation of the expected areas of interactions during human–robot navigation with respect to fuzzy and noisy information. The expected crossing points of the possible trajectories are nonlinearly associated with the positions and orientations of the robots and humans. The nonlinear transformation of a noisy system input, such as the directions of the motion of humans and robots, to a system output, the expected area of intersection of their trajectories, is performed by two methods: statistical linearization and the sigma-point transformation. For both approaches, fuzzy approximations are presented and the inverse problem is discussed where the input distribution parameters are computed from the given output distribution parameters.

## 1. Introduction

The planning and performing of mobile robot tasks in the presence of human operators while sharing the same workspace requires a high level of stability and safety. Research activities regarding navigation, obstacle avoidance, adaptation and collaboration between robots and human agents have been widely reported [1,2]. Multiple target tracking for robots using higher control levels in a control hierarchy are discussed in [3,4]. A human-friendly interaction between robots and humans can be obtained by human-like sensor systems [5]. A prominent role in robot navigation is the trajectory-crossing problem of robots and humans [6,7] and corresponding fuzzy solutions [8]. Motivations for a fuzzy solution of the intersection problem are manifold. One point is an uncertain measurement of the position and orientation of the human agent, because of which the use of a fuzzy signal and an adequate fuzzy processing seems natural [9,10]. Another aspect is the need for decreasing the computing effort in the case of complex calculations during a very small time interval. System uncertainties and observation noise lead to uncertainties of the intersection estimations.

The objective of this work is the formulation of the crossing/intersection problem by taking into account the uncertainties in human–robot systems, including sensors and motor characteristics. An important aspect is to define permissible uncertainties in a human–robot system for a given uncertainty at a possible intersection of their trajectories. Taking into account the nonlinearities, this is performed by the differential approach and a following analysis of the regarding Gaussian distributions. This approach is compared with the sigma-point transformation, which represents a simplification of the computation and a qualitative extension of the analysis regarding the statistics of the random variables. For broader areas of possible intersections, both methods are extended to fuzzy regions together with different numbers and shapes of fuzzy sets. The most important contributions are as follows:

- An investigation of uncertainties of possible intersection areas originating from sensor noise or system uncertainties.
- A direct and inverse transformation of the error variables at the intersection areas for two input variables (orientation angles) and two output variables (intersection coordinates).
- An extension of the method from two to six input variables (two orientation angles and four position coordinates).
- An exploration of the formulations of fuzzy versions.
- A formulation of the problem by the sigma-point transformation and corresponding comparison of the two methods.

This paper deals with the one-robot one-human trajectory-crossing problem, where small uncertainties in the position and orientation may lead to high uncertainties at the intersection points. The position and orientation of the human and robot are nonlinearly coupled but can be linearized. In the following, the linear part of the nonlinear system is considered in the analysis reported for small variations in the input [11]. Then, the “direct task” is described, meaning that the parameters of the input distribution are transformed to the output distribution parameters. The “inverse task” is also solved, meaning that for the defined output distribution parameters the input parameters are calculated. In this paper, two methods are outlined:

1. The *statistical linearization*, which linearizes the nonlinearity around the operating area at the intersection. The means and standard deviations on the input parameters positions (orientations) are transformed through the linearized nonlinear system to obtain the means and standard deviations of the output parameters (the position of intersection).
2. The *sigma-point transformation*, which calculates the so-called sigma points of the input distribution, including the mean and covariance of the input. The sigma points are directly propagated through the nonlinear system [12,13,14] to obtain the means and covariance of the output and, with this, the standard deviations of the output (the position of intersection). The advantage of the sigma-point transformation is that it captures the first- and second-order statistics of a random variable, whereas the statistical linearization approximates a random variable only by its first order. However, the computational complexity of the extended Kalman filter (EKF, differential approach) and unscented Kalman filter (UKF, sigma-point approach) is of the same order [13].

This paper is organized as follows. Section 2 describes the related work already conducted on unscented Kalman filters in mobile robot applications. In Section 3, the general intersection problem and its analytical approach is described. Section 4 deals with the transformation/conversion of Gaussian distributions for a two-input–two-output system and for a six-input–two-output system plus the corresponding inverse and fuzzy solutions. In Section 5, the sigma-point approach plus inverse and fuzzy solutions are addressed. Section 6 presents simulations of the *statistical linearization* and the *sigma-point transformation* to show the quality of the input–output conversion of the distributions and the impact of different resolutions of fuzzy approximations on the accuracy of the random variable intersection. Finally, Section 7 concludes this paper with a discussion of the two different approaches and a comparison of the methods.

## 2. Related Work

The crossing problem for mobile robots has been especially dealt with by [6,7]. Both publications deal with the so-called rendezvous problem whereby the key point is the trajectory planning under time constraints, taking into account the dynamics of the contributing robots. Uncertainties of possible intersection areas that come from sensor noise or system uncertainties are not discussed deeply. A fuzzy-adaptive extended Kalman filter (FAEKF) for the real-time attitude estimation of a mobile robot is proposed in [15] where fuzzy IF–THEN rules-based adaption laws modify the noise covariance matrices of the filter. However, the use of unscented Kalman filters or sigma-point transformation has not been discussed. For the estimation of landmarks, a simultaneous localization and mapping (SLAM) method is presented by [16] where an iterated sigma-point FastSLAM (ISP-FastSLAM) algorithm is proposed to minimize statistical linearization errors through the Gaussian–Newton iteration. A further application is presented by [17] where a walking robot uses sigma-point transformation for state estimation to guarantee stability in the system’s hybrid dynamics, which contains continuous and switching parts during movement. In [18], a vision-based SLAM system uses both extended Kalman filters (EKFs) and sigma-point Kalman filter (SPKF) algorithms and showed its superiority over the EKF. The pose estimation of mobile robots is discussed in [19] whereby several filter techniques like the Kalman filter (EKF), the unscented Kalman filter (UKF) and several variants of the particle filter (PF) are compared. It turns out that the UKF (also the sigma-point approach) exhibits almost the same computational cost. In [20], the inter-robot and robot–target correlations are discussed, and unscented transformation-based collaborative self-localization and a target tracking algorithm between robots are proposed. A tutorial on different approaches to exploit the structure of a system’s state and measurement models to reduce the computational demand of the algorithms is presented by [21]. In this publication, the computational complexity of different state estimation algorithms is presented, showing the superiority of the sigma-point transformation algorithms.

In all these publications, the problem of obstacle avoidance and/or the crossing problem in the presence of human actors are not taken into account, because of which the present paper is a further contribution to the robot–human interaction problem.

## 3. Computation of Intersections

The problem can be stated as follows:

A robot and human agent move in a common area according to their tasks or intentions. To avoid collisions, possible intersections of the paths of the agents should be predicted for both the trajectory planning and on-line interactions. To accomplish this, the positions, orientations and intended movements of the robot and human should be estimated as accurately as needed.

In this connection, uncertainties and noise on the random variables’ position/orientation $x_{R}$, $x_{H}$, $\phi_{R}$ and $\phi_{H}$ of the robot and human have a great impact on the calculation of the expected intersection position $x_{c}$. The random variable $x_{c}$ is calculated as the crossing point of the extension of the orientation or velocity vectors of the robot and human, which may change during motion depending on the task and current interaction. The task is to calculate the intersection and its uncertainty in the presence of the known uncertainties of the acting agent robot and human.

**Figure 1:**
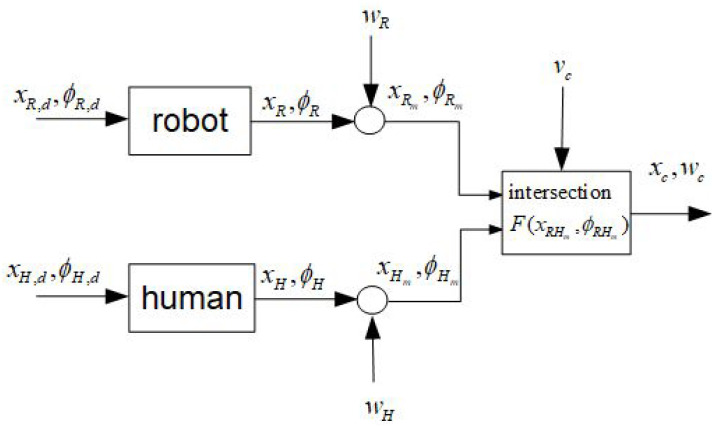
Intersection principle.

System noise $w_{R}$ and $w_{H}$ for the robot and human can be obtained from experiments. The noise $w_{c}$ of the “virtual” intersection is composed of the nonlinear transformed noise $w_{R}$ and $w_{H}$ and some additional noise $v_{c}$ that may come from uncertainties of the nonlinear computation of the intersection position $x_{c}$ (see Figure 1). In the following, the geometrical relations are described as well as the fuzzy approximations and nonlinear transformations of the random variables $x_{R}$, $x_{H}$, $\phi_{R}$ and $\phi_{H}$.

### 3.1. Geometrical Relations

**Figure 2:**
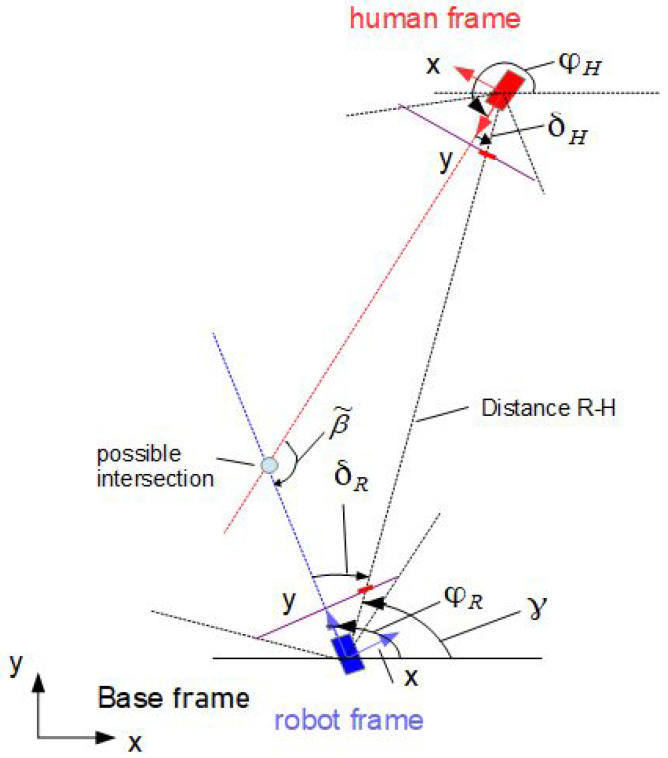
Human–robot scenario: geometry.

Let the y-axis of the mobile coordinate frame of the robot and human be aligned with their directions of motion. Furthermore, let the orientation angles $\phi_{R}$ and $\phi_{H}$ of the robot and human be measured from the x-axis of the base frame counterclockwise. Let the intersection $\left(x_{c},y_{c}\right)$ of the two linear trajectories $x_{R}\left(t\right)$ and $x_{H}\left(t\right)$ in a plane be described by the following relations (see Figure 2):

(1) $$\begin{array}{c} x_{H}=x_{R}+d_{RH}\cos\left(\phi_{R}+\delta_{R}\right)\\ y_{H}=y_{R}+d_{RH}\sin\left(\phi_{R}+\delta_{R}\right)\\ x_{R}=x_{H}+d_{RH}\cos\left(\phi_{H}+\delta_{H}\right)\\ y_{R}=y_{H}+d_{RH}\sin\left(\phi_{H}+\delta_{H}\right) \end{array}$$

where $x_{H}=\left(x_{H},y_{H}\right)$ and $x_{R}=\left(x_{R},y_{R}\right)$ are the positions of the human and robot and $\phi_{H}$ and $\phi_{R}$ are their orientation angles, and $\delta_{H}$ and $\delta_{R}$ are the positive angles measured from the *y* coordinates counterclockwise. The angle at the intersection is $\tilde{\beta }=\pi -\delta_{R}-\delta_{H}$. The variables $x_{H}$, $x_{R}$, $\phi_{R}$, $\phi_{H}$$\delta_{H}$ and $\phi_{H}$$\delta_{R}$; distance $d_{RH}$; and angle γ are assumed to be measurable. Angle γ is a bearing angle for the robot-to-human direction measured in base coordinates. If $\phi_{H}$ is not directly measurable, then it can be computed by

(2) $$\phi_{H}=\arcsin\left(\left(y_{H}-y_{R}\right)/d_{RH}\right)-\delta_{H}+\pi$$

The coordinates $x_{c}$ and $y_{c}$ of the intersection are computed straightforwardly by [8]

(3) $$\begin{array}{ccc} x_{c} & = & \frac{A-B}{\tan\phi_{R}-\tan\phi_{H}}\\ y_{c} & = & \frac{A\tan\phi_{H}-B\tan\phi_{R}}{\tan\phi_{R}-\tan\phi_{H}}\\ A & = & x_{R}\tan\phi_{R}-y_{R}\\ B & = & x_{H}\tan\phi_{H}-y_{H} \end{array}$$

Rewriting (3) leads to

(4) $$\begin{array}{ccc} x_{c} & = & \left(x_{R}\frac{\tan\phi_{R}}{G}-y_{R}\frac{1}{G}\right)-\left(x_{H}\frac{\tan\phi_{H}}{G}-y_{H}\frac{1}{G}\right)\\ y_{c} & = & \left(x_{R}\frac{\tan\phi_{R}\tan\phi_{H}}{G}-y_{R}\frac{\tan\phi_{H}}{G}\right)\\ & - & \left(x_{H}\frac{\tan\phi_{H}\tan\phi_{R}}{G}-y_{H}\frac{\tan\phi_{R}}{G}\right)\\ G & = & \tan\phi_{R}-\tan\phi_{H} \end{array}$$

After rearranging (4), we observe that $x_{c}={\left(x_{c},y_{c}\right)}^{T}$ is linear in $x_{RH}={\left(x_{R},y_{R},x_{H},y_{H}\right)}^{T}$

(5) $$\begin{array}{c} x_{c}=A_{RH}\cdot x_{RH} \end{array}$$

where

$$\begin{array}{c} A_{RH}=f\left(\phi_{R},\phi_{H}\right)=\;\;\;\;\;\\ \frac{1}{G}\left(\begin{array}{cccc} \tan\phi_{R} & -1 & -\tan\phi_{H} & 1\\ \tan\phi_{R}\tan\phi_{H} & -\tan\phi_{H} & -\tan\phi_{R}\tan\phi_{H} & \tan\phi_{R} \end{array}\right) \end{array}$$

This notation is of advantage for further computations, such as the fuzzification of the intersection problem and the transformation of the error distributions.

### 3.2. Computation of Intersections—Fuzzy Approach

The fuzzy solution presented in the following is a combination of classical analytical (crisp) methods and rule-based methods in the sense of a Takagi–Sugeno fuzzy rule base. An appropriate choice of the number of fuzzy sets and corresponding fuzzy rules depends strongly on the specific application. In the present case, fuzzy sets are used as the approximation of nonlinear functions. In the following, we introduce a fuzzy rule-based approximation of (5) with n×n fuzzy rules $R_{i,j}$

(6) $$\begin{array}{ccc} R_{i,j}:\;IF\;\phi_{R} & = & {\Phi_{R}}_{i}\;AND\;\phi_{H}={\Phi_{H}}_{j}\;\\ THEN\;x_{c} & = & {A_{RH}}_{i,j}\cdot x_{RH} \end{array}$$

*n*—the number of fuzzy terms, $\Phi_{R_{i}}$ and $\Phi_{H_{j}}$ for $\phi_{R}$ and $\phi_{H}$, with the result

(7) $$\begin{array}{c} x_{c}=\sum_{i,j}w_{i}\left(\phi_{R}\right)w_{j}\left(\phi_{H}\right)\cdot {A_{RH}}_{i,j}\cdot x_{RH} \end{array}$$

i,j=1…n, $w_{i}\left(\phi_{R}\right),w_{j}\left(\phi_{H}\right)\in \left[0,1\right]$ are normalized membership functions with $\sum_{i}w_{i}\left(\phi_{R}\right)=1$ and $\sum_{j}w_{j}\left(\phi_{H}\right)=1$.

**Figure 3:**
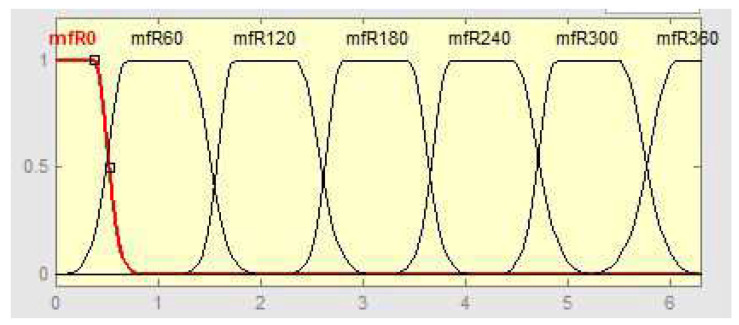
Membership functions for $\Delta \phi_{R},\Delta \phi_{H}=0-360^{{}^{\circ}}$.

**Figure 4:**
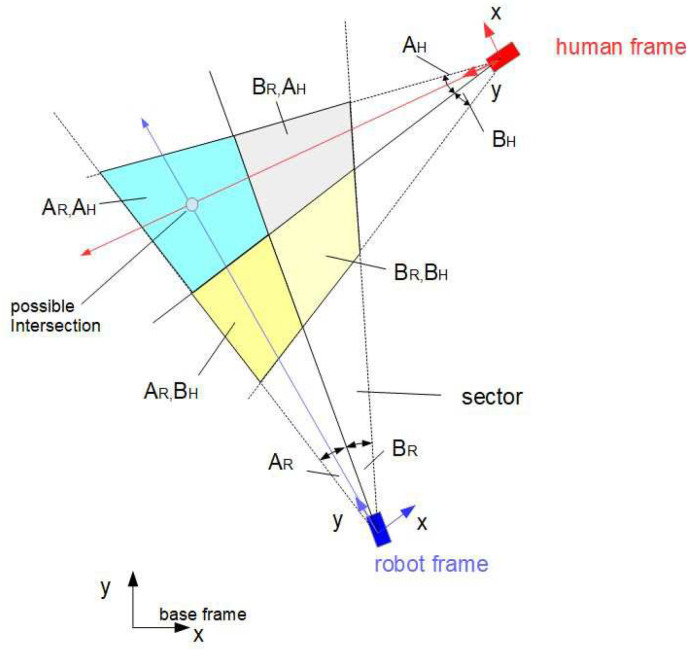
Fuzzy sectors.

Let the universes of discourse for $\phi_{R}$ and $\phi_{H}$ be $\phi_{R},\phi_{H}\in \left[0,360\right]$. Furthermore, let these universes of discourse be divided into *n* partitions (for example, 6) of 60, which leads to 6×6 fuzzy rules. The corresponding membership functions are shown in Figure 3. It turns out that this resolution leads to a poor fuzzy approximation. The approximation quality can be improved by increasing the number of fuzzy sets, which however results in a quadratic increase in the number of fuzzy rules. To avoid an “explosion” of the number of fuzzy rules being computed in one time step, a set of sub-areas covering a small number of rules for each sub-area is defined. Based on the measurements of $\phi_{R}$ and $\phi_{H}$, the appropriate sub-area is selected together with a corresponding set of rules (see Figure 4, sub-area $A_{R},A_{H}$). With this, the number of rules to be activated at one time step of calculation is low, although the total number of rules can be high. At the borderlines between the sub-areas, abrupt changes may occur, which can be avoided by overlapping the sub-areas.

### 3.3. Differential Approach

The positions and orientations of robots and humans are usually corrupted with noise originated from system uncertainties, sensor errors and motor characteristics. These uncertainties become apparent in uncertainties in the crossing/intersection areas of the trajectories of the robot and human. The analysis of uncertainty and noise at $x_{c}$ generated by the noise at $\phi_{R}$, $\phi_{H}$ and $x_{RH}={\left(x_{R},y_{R},x_{H},y_{H}\right)}^{T}$ requires a linearization of (4) around the operating points and with this a differential strategy. Let, for simplification, only the orientation angles $\phi_{R}$ and $\phi_{H}$ be corrupted with noise. In Section 4.3, the positions $x_{RH}={\left(x_{R},y_{R},x_{H},y_{H}\right)}^{T}$ are taken into account, too.

Differentiating (4) with $x_{RH}=const.$ yields

(8) $$\begin{array}{c} {\mathrm{dx}}_{c}=\tilde{J}\cdot dŒ\;\;\;\\ dŒ={\left(d\phi_{R}\;d\phi_{H}\right)}^{T};\;\tilde{J}=\left(\begin{array}{cc} {\tilde{J}}_{11} & {\tilde{J}}_{12}\\ {\tilde{J}}_{21} & {\tilde{J}}_{22} \end{array}\right) \end{array}$$

where

$$\begin{array}{ccc} {\tilde{J}}_{11} & = & \left(\begin{array}{cccc} -\tan\phi_{H} & 1 & \tan\phi_{H} & -1 \end{array}\right)\frac{x_{RH}}{G^{2}\cdot \cos^{2}\phi_{R}}\\ {\tilde{J}}_{12} & = & \left(\begin{array}{cccc} \tan\phi_{R} & -1 & -\tan\phi_{R} & 1 \end{array}\right)\frac{x_{RH}}{G^{2}\cdot \cos^{2}\phi_{H}}\\ {\tilde{J}}_{21} & = & {\tilde{J}}_{11}\cdot \tan\phi_{H}\\ {\tilde{J}}_{22} & = & {\tilde{J}}_{12}\cdot \tan\phi_{R} \end{array}$$

**Figure 5:**
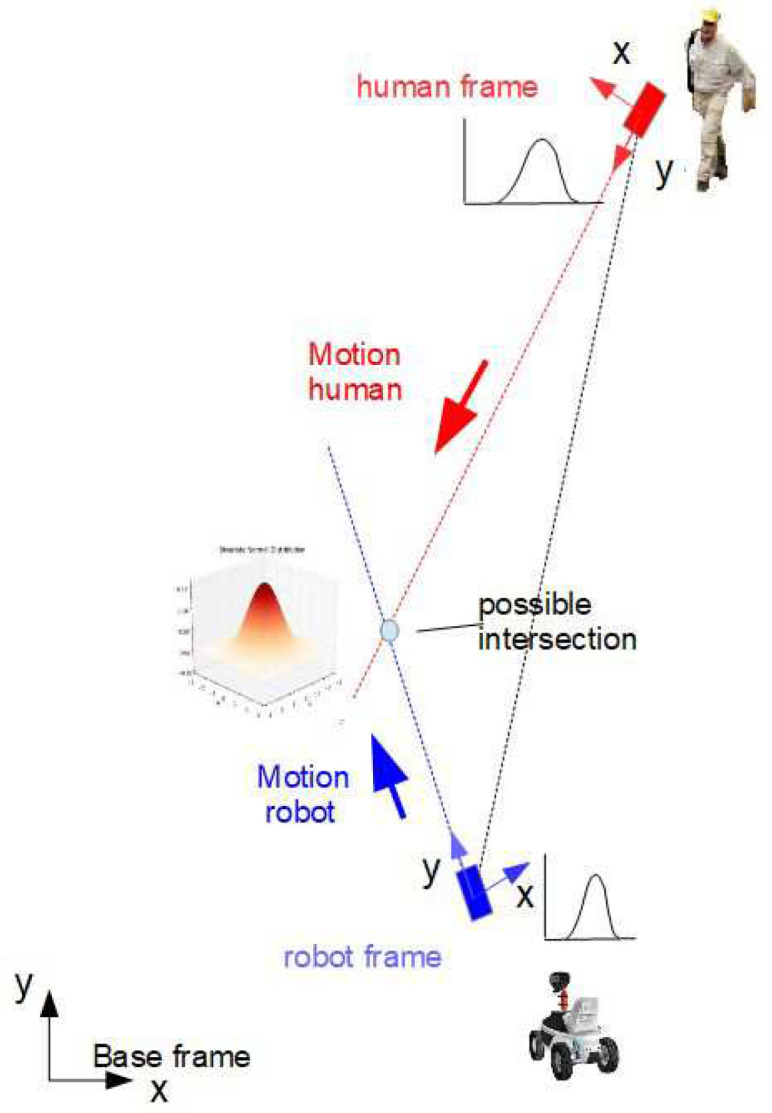
Intersection with noisy orientations.

The following sections deal with the accuracy of the computed intersection in the case of noisy orientation information (see Figure 5).

## 4. Transformation of Gaussian Distributions

### 4.1. General Assumptions

Consider a nonlinear system

(9) z=F(x)

where the random variables $x={\left(x_{1},x_{2}\right)}^{T}$ denote the input, $z={\left(z_{1},z_{2}\right)}^{T}$ denotes the output and *F* denotes a nonlinear transformation. The distribution of the uncorrelated Gaussian distributed components $x_{1}$ and $x_{2}$ is described by

(10) $$f_{x_{1},x_{2}}=\frac{1}{2\pi \sigma_{x_{1}}\sigma_{x_{2}}}exp\left(-\frac{1}{2}\left(\frac{e_{x_{1}}^{2}}{\sigma_{x_{1}}^{2}}+\frac{e_{x_{2}}^{2}}{\sigma_{x_{2}}^{2}}\right)\right)$$

where $e_{x_{1}}=x_{1}-\bar{x_{1}}$, with $\bar{x_{1}}$—the mean $\left(x_{1}\right)$ and $\sigma_{x_{1}}$—the standard deviation $x_{1}$, and $e_{x_{2}}=x_{2}-\bar{x_{2}}$, with $\bar{x_{2}}$—the mean $\left(x_{2}\right)$ and $\sigma_{x_{2}}$—the standard deviation $x_{2}$.

The goal is as follows: Given the nonlinear transformation (9) and the distribution (10), compute the output signals $z_{1}$ and $z_{2}$ and their distributions together with their standard deviations and the correlation coefficient. Linear systems transform Gaussian distributions linearly such that the output signals are also Gaussian-distributed. This does not apply for nonlinear systems, but if the input standard deviation is small enough, then a local linear transfer function can be built for which the outputs are Gaussian-distributed. Suppose the input standard deviations are small with respect to the nonlinear function, then the output distribution can be written as follows:

(11) $$\begin{array}{c} f_{z_{1},z_{2}}=\frac{1}{2\pi \sigma_{z_{1}}\sigma_{z_{2}}\sqrt{1-\rho_{z_{12}}^{2}}}\cdot \;\;\;\;\\ exp(-\frac{1}{2(1-\rho_{z_{12}}^{2})}\left(\frac{e_{z_{1}}^{2}}{\sigma_{z_{1}}^{2}}+\frac{e_{z_{2}}^{2}}{\sigma_{z_{2}}^{2}}-\frac{2\rho_{z_{12}}e_{z_{1}}e_{z_{2}}}{\sigma_{z_{1}}\sigma_{z_{2}}}\right)) \end{array}$$

$\rho_{z_{12}}$—the correlation coefficient.

### 4.2. Statistical Linearization, Two Inputs–Two Outputs

**Figure 6:**
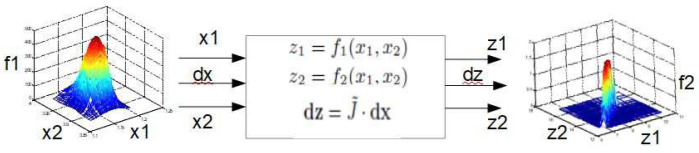
Differential transformation.

Let the nonlinear transformation F be described by two smooth transfer functions (see block scheme Figure 6)

(12) $$\begin{array}{c} z_{1}=f_{1}\left(x_{1},x_{2}\right)\\ z_{2}=f_{2}\left(x_{1},x_{2}\right) \end{array}$$

where $\left(x_{1},x_{2}\right)=\left(\phi_{R},\phi_{H}\right)$ and $\left(z_{1},z_{2}\right)=\left(x_{c},y_{c}\right)$.

The linearization of (12) yields

(13) $$\begin{array}{c} \mathrm{dz}=\tilde{J}\cdot \mathrm{dx}\;or\;e_{z}=\tilde{J}\cdot e_{x} \end{array}$$

with

(14) $$\begin{array}{c} e_{z}={\left(e_{z_{1}},e_{z_{2}}\right)}^{T}\;and\;e_{x}={\left(e_{x_{1}},e_{x_{2}}\right)}^{T}\\ \mathrm{dz}={\left(dz_{1},dz_{2}\right)}^{T}\;and\;\mathrm{dx}={\left(dx_{1},dx_{2}\right)}^{T} \end{array}$$

(15) $$\begin{array}{c} \tilde{J}=\left(\begin{array}{c} \partial f_{1}/\partial x_{1},\partial f_{1}/\partial x_{2}\\ \partial f_{2}/\partial x_{1},\partial f_{2}/\partial x_{2} \end{array}\right) \end{array}$$

#### 4.2.1. Output Distribution

To obtain the density $f_{z_{1},z_{2}}$ (11) of the output signal, we invert (15) and substitute the entries of $e_{x}$ into (10). $\tilde{J}$ is invertible if it is positive definite with $|\tilde{J}|>0$. Otherwise, there exist singularities due to different constellations of the position vector $x_{RH}$ and/or the orientations $\phi_{R}$ and $\phi_{H}$. To find all the singularities requires a further analysis, which is not the content of this paper. However, a simple heuristic leads us to some obvious situations: If $\phi_{R}=\phi_{H}$ or $\phi_{R}=\phi_{H}+\pi$, then the human and robot would move in parallel either in the same or the opposite direction. On the other hand, one may also obtain diverging trajectories with no crossing.

(16) $$\begin{array}{c} e_{x}=J\cdot e_{z} \end{array}$$

with $J={\tilde{J}}^{-1}$ and

(17) $$\begin{array}{c} J=\left(\begin{array}{cc} J_{11} & J_{12}\\ J_{21} & J_{22} \end{array}\right)=\left(\begin{array}{c} j_{\mathrm{xz}}\\ j_{\mathrm{yz}} \end{array}\right) \end{array}$$

where $j_{\mathrm{xz}}=\left(J_{11},J_{12}\right)$ and $j_{\mathrm{yz}}=\left(J_{21},J_{22}\right)$. The entries $J_{ij}$ are the result of the inversion of $\tilde{J}$. From this substitution, we obtain

(18) $$\begin{array}{c} f_{x_{1},x_{2}}=K_{x_{1},x_{2}}\cdot \;\;\;\;\\ exp(-\frac{1}{2}\cdot {e_{z}}^{T}\cdot \left({j_{x_{1},z}}^{T},{j_{x_{2},z}}^{T}\right)\cdot S_{x}^{-1}\cdot \left(\begin{array}{c} j_{x_{1},z}\\ j_{x_{2},z} \end{array}\right)\cdot e_{z}) \end{array}$$

where $K_{x_{1},x_{2}}=\frac{1}{2\pi \sigma_{x_{1}}\sigma_{x_{2}}}$ and

(19) $$\begin{array}{c} S_{x}^{-1}=\left(\begin{array}{c} \frac{1}{\sigma_{x_{1}}^{2}},0\\ 0,\frac{1}{\sigma_{x_{2}}^{2}} \end{array}\right) \end{array}$$

The exponent of (18) is rewritten into

(20) $$\begin{array}{c} x_{po}=-\frac{1}{2}\cdot (\frac{1}{\sigma_{x_{1}}^{2}}{\left(e_{z_{1}}J_{11}+e_{z_{2}}J_{12}\right)}^{2}\\ +\frac{1}{\sigma_{x_{2}}^{2}}{\left(e_{z_{1}}J_{21}+e_{z_{2}}J_{22}\right)}^{2}) \end{array}$$

and furthermore

(21) $$\begin{array}{c} x_{po}=-\frac{1}{2}\cdot [e_{z_{1}}^{2}\left(\frac{J_{11}^{2}}{\sigma_{x_{1}}^{2}}+\frac{J_{21}^{2}}{\sigma_{x_{2}}^{2}}\right)+e_{z_{2}}^{2}\left(\frac{J_{12}^{2}}{\sigma_{x_{1}}^{2}}+\frac{J_{22}^{2}}{\sigma_{x_{2}}^{2}}\right)+\\ 2\cdot e_{z_{1}}e_{z_{2}}\left(\frac{J_{11}J_{12}}{\sigma_{x_{1}}^{2}}+\frac{J_{21}J_{22}}{\sigma_{x_{2}}^{2}}\right)]\;\; \end{array}$$

Let

(22) $$\begin{array}{c} A=\left(\frac{J_{11}^{2}}{\sigma_{x_{1}}^{2}}+\frac{J_{21}^{2}}{\sigma_{x_{2}}^{2}}\right);\;B=\left(\frac{J_{12}^{2}}{\sigma_{x_{1}}^{2}}+\frac{J_{22}^{2}}{\sigma_{x_{2}}^{2}}\right)\\ C=(\frac{J_{11}J_{12}}{\sigma_{x_{1}}^{2}}+\frac{J_{21}J_{22}}{\sigma_{x_{2}}^{2}})\;\; \end{array}$$

then a comparison of $x_{po}$ in (21) and the exponent in (11) yields

(23) $$\begin{array}{c} \frac{1}{\left(1-\rho_{z_{12}}^{2}\right)}\frac{1}{\sigma_{z_{1}}^{2}}=A;\;\frac{1}{\left(1-\rho_{z_{12}}^{2}\right)}\frac{1}{\sigma_{z_{2}}^{2}}=B\\ \frac{-2\rho_{z_{12}}}{\left(1-\rho_{z_{12}}^{2}\right)}\frac{1}{\sigma_{z_{1}}\sigma_{z_{2}}}=2C\;\; \end{array}$$

The standard deviations $\sigma_{z_{1}}$ and $\sigma_{z_{2}}$ and the correlation coefficient $\rho_{z_{12}}$ yield

(24) $$\begin{array}{c} \rho_{z_{12}}=-\frac{C}{\sqrt{AB}}\;\;\;\;\\ \frac{1}{\sigma_{z_{1}}^{2}}=A-\frac{C^{2}}{B};\;\frac{1}{\sigma_{z_{2}}^{2}}=B-\frac{C^{2}}{A} \end{array}$$

The result is as follows: If the parameter of the input distribution and the transfer function F(x,y) are known, then the output distribution parameters can be computed straightforwardly.

#### 4.2.2. Fuzzy Solution

To save computing costs in real time, we create a TS fuzzy model that is represented by the rules $R_{ij}$.

(25) $$\begin{array}{c} R_{ij}:\;\;\;\;\\ IF\;x_{1}={X_{1}}_{i}\;AND\;x_{2}={X_{2}}_{i} \end{array}$$

$$\begin{array}{c} THEN\;\rho_{z_{12}}=-\frac{C_{ij}}{\sqrt{A_{ij}B_{ij}}}\\ AND\;\;\;\;\;\\ \frac{1}{\sigma_{z_{1}}^{2}}=A_{ij}-\frac{C_{ij}^{2}}{B_{ij}};\\ AND\;\;\;\;\;\\ \frac{1}{\sigma_{z_{2}}^{2}}=B_{ij}-\frac{C_{ij}^{2}}{A_{ij}} \end{array}$$

where ${X_{1}}_{i},{X_{2}}_{i}$ are fuzzy terms for $x_{1},x_{2}$, and $A_{ij},B_{ij},C_{ij}$ are functions of the predefined variables $x_{1}={x_{1}}_{i}$ and $x_{2}={x_{2}}_{i}$.

From (25), we derive

(26) $$\begin{array}{c} \rho_{z_{12}}=-\sum_{ij}w_{i}\left(x_{1}\right)w_{j}\left(x_{2}\right)\frac{C_{ij}}{\sqrt{A_{ij}B_{ij}}}\\ \frac{1}{\sigma_{z_{1}}^{2}}=\sum_{ij}w_{i}\left(x_{1}\right)w_{j}\left(x_{2}\right)\left(A_{ij}-\frac{C_{ij}^{2}}{B_{ij}}\right)\\ \frac{1}{\sigma_{z_{2}}^{2}}=\sum_{ij}w_{i}\left(x_{1}\right)w_{j}\left(x_{2}\right)\left(B_{ij}-\frac{C_{ij}^{2}}{A_{ij}}\right) \end{array}$$

$w_{i}\left(x_{1}\right)\in \left[0,1\right]$ and $w_{j}\left(x_{2}\right)\in \left[0,1\right]$ are the weighting functions with $\sum_{i}w_{i}\left(x_{1}\right)=1$, $\sum_{j}w_{j}\left(x_{2}\right)=1$.

#### 4.2.3. Inverse Solution

The previous paragraph discussed the direct transformation task: Let the distribution parameters of the input variable be defined and find the corresponding output parameters. However, it might also be useful to solve the inverse task: Given the output parameters (standard deviation and correlation coefficient), find the corresponding input parameters. This solution of the inverse task is similar to those discussed in Section 4.2. The starting points are equations (10) and (11), which describe the distributions of the inputs and outputs, respectively. Then, we substitute (13) into (10) and rename the resulting exponent ${x_{po}}_{z}$ into ${x_{po}}_{x}$ and discuss the exponent ${x_{po}}_{x}$

(27) $$\begin{array}{c} {x_{po}}_{x}=\frac{-1}{2(1-\rho_{z_{12}}^{2})}\left({e_{x}}^{T}{\tilde{J}}^{T}S_{z}^{-1}\tilde{J}e_{x}-\frac{2\rho_{z_{12}}e_{z_{1}}e_{z_{2}}}{\sigma_{z_{1}}\sigma_{z_{2}}}\right) \end{array}$$

with

$$\begin{array}{c} S_{x}^{-1}=\left(\begin{array}{c} \frac{1}{\sigma_{z_{1}}^{2}},0\\ 0,\frac{1}{\sigma_{z_{2}}^{2}} \end{array}\right) \end{array}$$

Now, comparing (27) with the exponent of (10) of the input density, we find that the mixed term in (27) must be zero, from which we obtain the correlation coefficient $\rho_{z_{12}}$ and with this the standard deviations of the inputs

(28) $$\begin{array}{c} \rho_{z_{12}}=\left(\frac{{\tilde{J}}_{11}{\tilde{J}}_{12}}{\sigma_{z_{1}}^{2}}+\frac{{\tilde{J}}_{21}{\tilde{J}}_{22}}{\sigma_{z_{2}}^{2}}\right)\frac{\sigma_{z_{1}}\sigma_{z_{2}}}{\left({\tilde{J}}_{11}{\tilde{J}}_{22}+{\tilde{J}}_{12}{\tilde{J}}_{21}\right)}\\ \frac{1}{\sigma_{x}^{2}}=\left(\frac{{\tilde{J}}_{11}^{2}}{\sigma_{z_{1}}^{2}}+\frac{{\tilde{J}}_{21}^{2}}{\sigma_{z_{2}}^{2}}-\frac{2\rho_{z_{12}}}{\sigma_{z_{1}}\sigma_{z_{2}}}{\tilde{J}}_{11}{\tilde{J}}_{21}\right)/\left(1-\rho_{z_{12}}^{2}\right)\\ \frac{1}{\sigma_{y}^{2}}=\left(\frac{{\tilde{J}}_{12}^{2}}{\sigma_{z_{1}}^{2}}+\frac{{\tilde{J}}_{22}^{2}}{\sigma_{z_{2}}^{2}}-\frac{2\rho_{z_{12}}}{\sigma_{z_{1}}\sigma_{z_{2}}}{\tilde{J}}_{12}{\tilde{J}}_{22}\right)/\left(1-\rho_{z_{12}}^{2}\right) \end{array}$$

The detailed development can be found in [22].

### 4.3. Six Inputs–Two Outputs

Consider again the nonlinear system

(29) $$x_{c}=F\left(x\right)$$

In the previous subsections, we assumed the positions $x_{R}$ and $x_{H}$ not to be corrupted with noise. However, taking into account the positions to be random variables, the number of inputs is 6 so that the input vector yields $x={\left(x_{1},x_{2},x_{3},x_{4},x_{5},x_{6}\right)}^{T}$ or $x=(\phi_{R},\phi_{H},x_{R},y_{R},x_{H},y_{H})$ with the output vector $x_{c}={\left(x_{c},y_{c}\right)}^{T}$.

Furthermore, let the uncorrelated Gaussian-distributed inputs $x_{1}\ldots x_{6}$ be described by the 6-dim density

(30) $$f_{x_{i}}=\frac{1}{{\left(2\pi \right)}^{6/2}{\left|S_{x}\right|}^{1/2}}exp\left(-\frac{1}{2}\left({e_{x}}^{T}{S_{x}}^{-1}e_{x}\right)\right)$$

where $e_{x}={\left({e_{x}}_{1},{e_{x}}_{2},\ldots ,{e_{x}}_{6}\right)}^{T}$; $e_{x}=x-\bar{x}$, $\bar{x}$—the mean(x) and $S_{x}$—the covariance matrix.

$$S_{x}=\left(\begin{array}{cccc} \sigma_{x_{1}}^{2} & 0 & \ldots & 0\\ 0 & \sigma_{x_{2}}^{2} & \ldots & 0\\ \ldots & \ldots & \ldots & \ldots \\ 0 & \ldots & 0 & \sigma_{x_{6}}^{2} \end{array}\right)$$

According to (11), the output density is described by

(31) $$\begin{array}{c} f_{x_{c},y_{c}}=\frac{1}{2\pi \sigma_{x_{c}}\sigma_{y_{c}}\sqrt{1-\rho^{2}}}\cdot \\ exp(-\frac{1}{2(1-\rho^{2})}\left(e_{x_{c}}^{T}{S_{c}}^{-1}e_{x_{c}}-\frac{2\rho e_{x_{c}}e_{y_{c}}}{\sigma_{x_{c}}\sigma_{y_{c}}}\right)) \end{array}$$

ρ—the correlation coefficient, $e_{x_{c}}={\left(e_{x_{c}},e_{y_{c}}\right)}^{T}$.

After some calculations [23], we find for ρ, $\frac{1}{\sigma_{x_{c}}^{2}}$ and $\frac{1}{\sigma_{y_{c}}^{2}}$

(32) $$\begin{array}{ccc} \rho & = & -\frac{C}{\sqrt{AD}}\\ \frac{1}{\sigma_{x_{c}}^{2}} & = & A-\frac{C^{2}}{D};\;\frac{1}{\sigma_{y_{c}}^{2}}=D-\frac{C^{2}}{A} \end{array}$$

with

(33) $$\begin{array}{c} A=\sum_{i=1}^{6}\frac{1}{\sigma_{x_{i}}^{2}}J_{i1}^{2};\;B=\sum_{i=1}^{6}\frac{1}{\sigma_{x_{i}}^{2}}J_{i1}J_{i2}\\ C=\sum_{i=1}^{6}\frac{1}{\sigma_{x_{i}}^{2}}J_{i1}J_{i2};\;D=\sum_{i=1}^{6}\frac{1}{\sigma_{x_{i}}^{2}}J_{i2}^{2} \end{array}$$

This is the counterpart to the 2-dim input case (24).

#### 4.3.1. Inverse Solution

An inverse solution cannot be uniquely computed due to the undetermined character of the 6-input–2-output system. Therefore, from the required variances at the intersection position (output), the corresponding variances for the positions and orientations of the robot–human or robot–robot (input) cannot be concluded.

#### 4.3.2. Fuzzy Approach

The steps to the fuzzy approach are very similar to those of the 2-input case:

- Define the operation points $x_{i}={\left(x_{1},x_{2},x_{3},x_{4},x_{5},x_{6}\right)}_{i}^{T}$;
- Compute $A_{i}$, $B_{i}$ and $C_{i}$ at $x_{i}={\left(x_{1},x_{2},x_{3},x_{4},x_{5},x_{6}\right)}_{i}^{T}$ from (33);
- Formulate the fuzzy rules $R_{i}$ according to (25) and (26), i=1…n.

The number *n* of rules is computed as follows:

With l=6—the number of fuzzy terms and k=6—the number of inputs, we obtain $n=l^{k}=6^{6}$—the number of rules.

This number of rules is unacceptably high. To limit *n* to an adequate number, one has to limit the number of inputs and/or fuzzy terms to look for the most influential variables either in a heuristic or systematic way [24]. This however is not the issue to be discussed in this paper.

## 5. Sigma-Point Transformation

In the following, the estimation/identification of the standard deviations of possible intersection coordinates of trajectories for both the robot–robot and human–robot combinations by means of the sigma-point technique is discussed. The following method is based on the unscented Kalman filter technique where the intersections cannot be directly measured but predicted/computed only. Nevertheless, it is possible to compute the variance of the predicted events, such as possible collisions or planned rendezvous situations, by a direct propagation of statistical parameters—the sigma points—through the nonlinear geometrical relation, which is a result of the crossing of two trajectories. Let $x={\left(x_{1},x_{2}\right)}^{T}$—the input vector and $x_{c}={\left(x_{c1},x_{c2}\right)}^{T}$—the output vector where for the special case ${\left(x_{1},x_{2}\right)}^{T}={\left(\phi_{R},\phi_{H}\right)}^{T}$ and ${\left(x_{c1},x_{c2}\right)}^{T}={\left(x_{c},y_{c}\right)}^{T}$. The nonlinear relation between x and $x_{c}$ is given by (34)

(34) $$x_{c}=F(x)$$

For the discrete case, we obtain for the state $x_{c}$

(35) $$x_{c}\left(k\right)=F\left(x\left(k-1\right)+w\left(k-1\right)\right)$$

and for the measured output $z_{c}\left(k\right)$

(36) $$z_{c}\left(k\right)=h(x_{c})\left(k\right)+v\left(k\right))$$

where w and v are the system noise and measurement noise, respectively. $h(x_{c})$ is the output nonlinearity. Furthermore, let there be the following:

$\bar{x}\left(k\right)$—the mean at time $t_{k}$;
P(k)—the covariance matrix;
$x_{0}$—the initial state with the known mean $\mu_{0}=E\left(x_{0}\right)$;
$P_{0}\left(k\right)=E\left[\left(x_{0}-\mu_{0}\right){\left(x_{0}-\mu_{0}\right)}^{T}\right]$.

### 5.1. Selection of Sigma Points

**Figure 7:**
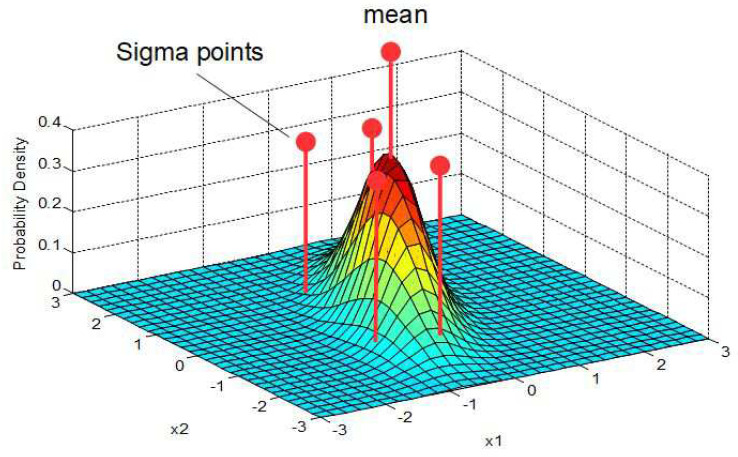
Sigma points for a 2-dim Gaussian random variable.

Sigma points are the selected parameters of a given error distribution of a random variable. Sigma points lie along the major eigen-axes of the covariance matrix of the random variable. The height of each sigma point (see Figure 7) represents its relative weight $W^{j}$ used in the following selection procedure.

Let X(k−1) be a set of 2n+1 sigma points where *n* is the dimension of the state space (in our example, n=2).

(37) $$X\left(k-1\right)=\{\left(x^{j}\left(k-1\right),W^{j}\right)|j=0\ldots 2n\}$$

Consider the following selection of sigma points

(38) $$\begin{array}{ccc} x^{0}\left(k-1\right) & = & \bar{x}\left(k-1\right)\\ & & -1<W^{0}<1\\ W^{0} & = & \frac{\lambda }{n+\lambda };\;\lambda =\alpha^{2}\left(n+\kappa \right)-n \end{array}$$

$$\begin{array}{cccc} & x^{i}\left(k-1\right) & = & \bar{x}\left(k-1\right)+\sqrt{\left(\frac{n}{1-W^{0}}P\left(k-1\right)\right)};\;i=1\ldots n\\ & x^{i}\left(k-1\right) & = & \bar{x}\left(k-1\right)-\sqrt{\left(\frac{n}{1-W^{0}}P\left(k-1\right)\right)};\;i=\left(n+1\right)\ldots 2n \end{array}$$

(39) $$\begin{array}{ccc} W^{j} & = & \frac{1-W^{0}}{2n} \end{array}$$

under the following condition

(40) $$\sum_{j=0}^{2n}W^{j}=1$$

α and κ are scaling factors. A usual choice is $\alpha =10^{-2}$ and κ=0. $\sqrt{\frac{n}{1-W^{0}}P\left(k-1\right)}$ is the row/column of the matrix square root of $\frac{n}{1-W^{0}}P$. The square root of a matrix P is the solution S for P=S·S, which is obtained by Cholesky factorization.

### 5.2. Model Forecast Step

To go on with the UKF, the following step is devoted to the model forecast. In this way, the sigma points $x^{j}\left(k\right)$ are propagated through the nonlinear process model

(41) $$x_{c}^{f,j}\left(k\right)=F\left(x^{j}\left(k-1\right)\right)$$

where the superscript *f* means “forecast”. From these transformed and forecasted sigma points, the mean and covariance for the forecast value of $x_{c}\left(k\right)$ are

(42) $$\begin{array}{ccc} & x_{c}^{f}\left(k\right) & =\sum_{j=0}^{2n}W^{j}x_{c}^{f,j}\left(k\right)\\ & P^{f}\left(k\right) & =\sum_{j=0}^{2n}W^{j}\left(x_{c}^{f,j}\left(k\right)-x_{c}^{f}\left(k\right)\right){\left(x_{c}^{f,j}\left(k\right)-x_{c}^{f}\left(k\right)\right)}^{T} \end{array}$$

### 5.3. Measurement Update Step

In this step, the sigma points are propagated through the nonlinear observation model

(43) $$z_{c}^{f,j}\left(k\right)=h\left(x_{c}^{j}\left(k-1\right)\right)$$

from which we obtain the mean and covariance (innovation covariance)

(44) $$\begin{array}{c} z_{c}^{f}\left(k-1\right)=\sum_{j=0}^{2n}W^{j}z_{c}^{f,j}\left(k-1\right)\\ Cov({\tilde{z}}_{c}^{f}\left(k-1\right))=\\ \sum_{j=0}^{2n}W^{j}\left(z_{c}^{f,j}\left(k-1\right)-z_{c}^{f}\left(k-1\right)\right)\times \\ {\left(z_{c}^{f,j}\left(k-1\right)-z_{c}^{f}\left(k-1\right)\right)}^{T}+R\left(k\right) \end{array}$$

and the cross-covariance

(45) $$\begin{array}{c} Cov({\tilde{x}}_{c}^{f}\left(k\right),{\tilde{z}}_{c}^{f}\left(k-1\right))=\\ \sum_{j=0}^{2n}W^{j}\left(x_{c}^{f,j}\left(k\right)-x_{c}^{f}\left(k\right)\right){\left(z_{c}^{f,j}\left(k-1\right)-z_{c}^{f}\left(k-1\right)\right)}^{T} \end{array}$$

### 5.4. Data Assimilation Step

In this step, the forecast information is combined with the new information from the output z(k) from which we obtain, with the Kalman filter, gain K

(46) $${\hat{x}}_{c}\left(k\right)=x_{c}^{f}\left(k\right)+K\left(k\right)\left(z_{c}\left(k\right)-z_{c}^{f}\left(k-1\right)\right)$$

The gain K is given by

(47) $$K\left(k\right)=Cov\left({\tilde{x}}_{c}^{f}\left(k\right),{\tilde{z}}_{c}^{f}\left(k-1\right)\right)\cdot Cov^{-1}\left({\tilde{z}}_{c}^{f}\left(k-1\right)\right)$$

and the posterior covariance is updated by

(48) $$P\left(k\right)=P^{f}\left(k\right)-K\left(k\right)\cdot Cov\left({\tilde{z}}_{c}^{f}\left(k-1\right)\right)K^{T}\left(k\right)$$

Usually, it is sufficient to compute the mean and variance for the output/state $x_{c}$ of the nonlinear static system F(x). In this case, it is possible to stop further computing at Equation (42), meaning to rather calculate the transformed sigma points $x_{c}^{f,j}$ and develop the specific output means and variances from (41) and (42). In this connection, it is enough to substitute the covariance matrix Q into (38) instead of P. One advantage of the sigma-point approach prior to statistical linearization is the easy scalability to multi-dimensional random variables.

For the intersection problem, there are 2 cases:

1. The 2 inputs, 2 outputs (2 orientation angles and 2 crossing coordinates);
2. The 6 inputs, 2 outputs (2 orientation angles and 4 position coordinates, and 2 crossing coordinates).

For the statistical linearization (method 1), the step from the 2 inputs–2 outputs case to the (6,2)-case is computationally more costly than that for the sigma-point approach (method 2), (see Equations (20)–(24) versus Equations (37) and (40)–(42)).

### 5.5. Sigma Points—Fuzzy Solutions

In order to lower the computing effort, the application of the TS fuzzy interpolation may be a solution, which will be shown in the following. Having a look at the two-dimensional problem, we can see a nonlinear propagation of the input sigma points through a nonlinear function F. Let $x^{j}$ be the two-dimensional “input” sigma points

(49) $$x^{j}={\left(x_{1}^{j},x_{2}^{j}\right)}^{T}$$

or for the special case “intersection”

(50) $$x^{j}={\left(\phi_{R}^{j},\phi_{H}^{j}\right)}^{T}$$

The propagation through F leads to the “output” sigma points

(51) $$x_{c}^{f,j}\left(k\right)=F\left(x^{j}\left(k-1\right)\right)$$

or for the special case

(52) $$\begin{array}{ccc} x_{c}^{f,j}\left(k\right) & = & F(x_{1}^{j}\left(k-1\right),x_{2}^{j}\left(k-1\right))=\\ & & F(\phi_{R}^{j}\left(k-1\right),\phi_{H}^{j}\left(k-1\right)) \end{array}$$

The special nonlinear function F is described by (see (5))

(53) $$\begin{array}{c} x_{c}=A_{RH}\left(\phi_{R},\phi_{H}\right)\cdot x_{RH} \end{array}$$

where $A_{RH}$ is a nonlinear matrix (6) linearly combined with the position vector $x_{RH}={\left(x_{R},y_{R},x_{H},y_{H}\right)}^{T}$.

A fuzzification aims at $A_{RH}$:

(54) $$\begin{array}{c} F^{fuzz}\left(\phi_{R},\phi_{H}\right)=A_{RH}^{fuzz}\cdot x_{RH}=\\ \sum_{l_{1},l_{2}}^{m}w^{l_{1}}\left(\phi_{R}\right)w^{l_{2}}\left(\phi_{H}\right)\cdot A_{RH}\left(\phi_{R}^{l_{1}},\phi_{H}^{l_{2}}\right)\cdot x_{RH} \end{array}$$

Applied to the sigma points $\left(\phi_{R}^{j},\phi_{H}^{j}\right)$, we obtain a TS fuzzy model described by the following rules $R_{l_{1},l_{2}}$

(55) $$R_{l_{1},l_{2}}:$$

$$\begin{array}{c} IF\;\phi_{R}^{j}={\Phi_{R}^{j}}_{l_{1}}\;AND\;\phi_{H}^{j}={\Phi_{H}^{j}}_{l_{2}}\\ THEN\;x_{c}^{f,j}=A_{RH}\left(\phi_{R}^{l_{1},j},\phi_{H}^{l_{2},j}\right)\cdot x_{RH} \end{array}$$

where ${\Phi_{R}^{j}}_{l_{1}},{\Phi_{H}^{j}}_{l_{2}}$ are fuzzy terms for $\phi_{R}^{j},\phi_{H}^{j}$; the matrices $A_{RH}$ are functions of the predefined variables $\phi_{R}^{j}$ and $\phi_{H}^{j}$. This set of rules leads to the result

(56) $$\begin{array}{c} x_{c}^{f,j}=F^{fuzz}\left(\phi_{R}^{j},\phi_{H}^{j}\right)=\\ \sum_{l_{1},l_{2}}^{m}w^{l_{1}}\left(\phi_{R}^{j}\right)w^{l_{2}}\left(\phi_{H}^{j}\right)\cdot A_{RH}\left(\phi_{R}^{l_{1},j},\phi_{H}^{l_{2},j}\right)\cdot x_{RH} \end{array}$$

$w^{l_{1}}\left(\phi_{R}^{j}\right)\in \left[0,1\right]$ and $w^{l_{2}}\left(\phi_{H}^{j}\right)\in \left[0,1\right]$ are weighting functions with $\sum_{l_{1}}w^{l_{1}}=1$, $\sum_{l_{2}}w^{l_{2}}=1$. The advantage of this approach is that the $l_{1}\times l_{2}$ matrices $A_{RH}^{l_{1},l_{2},j}=A_{RH}\left(\phi_{R}^{l_{1},j},\phi_{H}^{l_{2},j}\right)$ can be computed off-line. Then, the calculation of the mean and covariance matrix is obtained by

(57) $$\begin{array}{c} x_{c}^{f}\left(k\right)=\sum_{j=0}^{2n}W^{j}x_{c}^{f,j}\left(k\right)\\ P^{f}\left(k\right)=\sum_{j=0}^{2n}W^{j}{\tilde{x}}_{c}^{f,j}\left(k\right){\left({\tilde{x}}_{c}^{f,j}\left(k\right)\right)}^{T}\\ {\tilde{x}}_{c}^{f,j}=x_{c}^{f,j}-x_{c}^{f} \end{array}$$

From the covariance $P^{f}$, the variances ${\sigma_{c}}_{xx},{\sigma_{c}}_{yy},{\sigma_{c}}_{xy}$ can be obtained

(58) $$\begin{array}{c} {\sigma_{c}}_{xx}=E\left({\left(x_{c}^{f}-{\bar{x}}_{c}^{f}\right)}^{2}\right))\\ {\sigma_{c}}_{yy}=E\left({\left(y_{c}^{f}-{\bar{y}}_{c}^{f}\right)}^{2}\right))\\ {\sigma_{c}}_{xy}={\sigma_{c}}_{yx}=E\left(\left(x_{c}^{f}-{\bar{x}}_{c}^{f}\right)\cdot \left(y_{c}^{f}-{\bar{y}}_{c}^{f}\right)\right) \end{array}$$

### 5.6. Inverse Solution

The inverse solution for the sigma-point approach is much easier to obtain than that for the statistical linearization method. Starting from Equation (34), we build the inverse function

(59) $$x=F^{-1}\left(x_{c}\right)$$

on the condition that $F^{-1}$ exists. Then, the covariance matrix P is defined in correspondence to the required variances ${\sigma_{c}}_{xx},{\sigma_{c}}_{yy}$ and ${\sigma_{c}}_{xy}$. The following steps correspond to Equations (34)–(42). The position vector $x_{RH}$ is assumed to be known. The inversion of F requires a linearization of $x_{RH}$ and a starting point to obtain a stable convergence to the inverse $F^{-1}$. The result is the mean x and the covariance Q at the input. A reliable inversion is only possible for the 2-input–2-output case.

### 5.7. Six-Inputs–Two-Outputs

This case works exactly as the 2-input–2-output case along with Equations (34)–(42) due to the fact that the computation of the sigma points (38)–(40) and the propagation through the nonlinearity F automatically include the input and output dimensions.

## 6. Simulation Results

The following simulations show the results of the uncertainties of the predicted intersections based on statistical linearization and sigma-point transformation. For both methods, identical parameters are employed for comparison reasons (see Figure 2). The position/orientation of the robot and human are given by the following:

$x_{R}={\left(x_{R},y_{R}\right)}^{T}={\left(2,0\right)}^{T}$m;
$x_{H}={\left(x_{H},y_{H}\right)}^{T}={\left(4,10\right)}^{T}$m;
$\phi_{R}=1.78$ rad = 102°;
$\phi_{H}=3.69$ rad = 212°.
$\phi_{R}$ and $\phi_{H}$ are corrupted by Gaussian noise with standard deviations (std) of $\sigma_{\phi_{R}}=\sigma_{x_{1}}=0.02$ rad, ${(=1.1}^{{}^{\circ}})$, and $\sigma_{\phi_{H}}=\sigma_{x_{2}}=0.02$ rad, ${(=1.1}^{{}^{\circ}})$.

### 6.1. Statistical Linearization

Table 1 shows a comparison of the non-fuzzy method with the fuzzy approach using sectors of $60^{{}^{\circ}},30^{{}^{\circ}},15^{{}^{\circ}},7.5^{{}^{\circ}}$ of the unit circle for the orientations of the robot and human. The notations in Table 2 are as follows: ${\sigma_{x}}_{c}$—std-computed, ${\sigma_{x}}_{m}$—std-measured, etc. As expected, we see that higher resolutions lead to a better match between the fuzzy and analytical approach. Furthermore, the match between the measured and calculated values depends on the form of membership functions (MFS). For example, low input standard deviations (0.02 rad) show a better match for Gaussian membership functions, and higher input standard deviations (0.05 rad = $2.9^{{}^{\circ}}$) require Gaussian bell-shaped membership functions, which comes from different smoothing effects (see columns 4 and 5 in Table 2).

**Figure 8:**
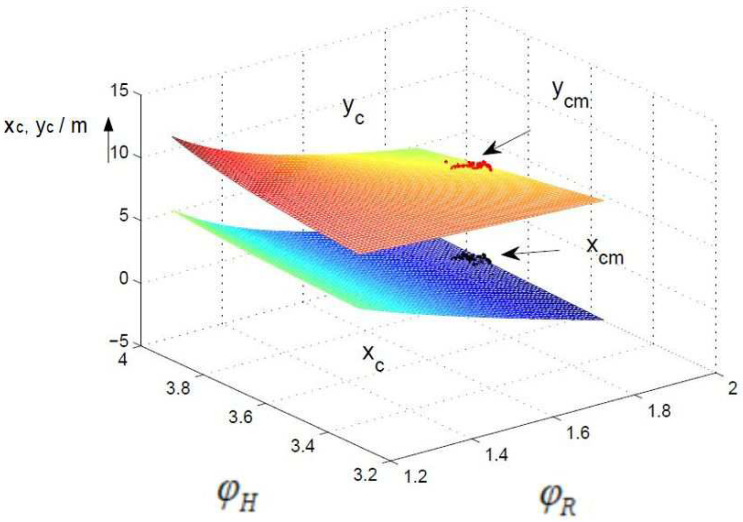
Control surface non-fuzzy, units of $\phi_{R}$ and $\phi_{H}$ in rad.

**Figure 9:**
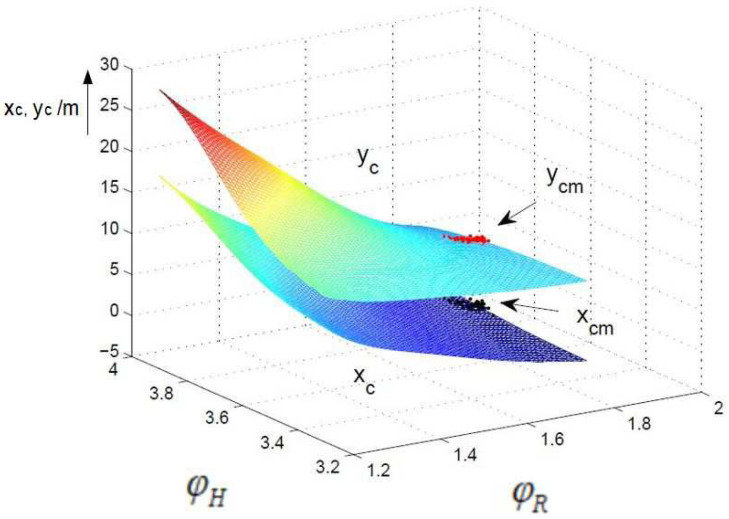
Control surface fuzzy, $30^{{}^{\circ}}$, units of $\phi_{R}$ and $\phi_{H}$ in rad.

**Figure 10:**
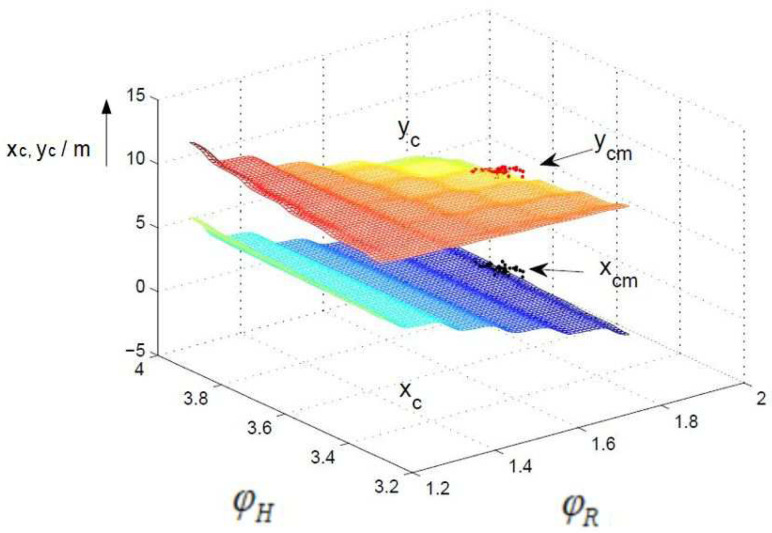
Control surface fuzzy, $7.5^{{}^{\circ}}$, units of $\phi_{R}$ and $\phi_{H}$ in rad.

A comparison of the control surfaces and corresponding measurements $x_{cm},y_{cm}$ (black and red dots) is depicted in Figure 8, Figure 9 and Figure 10. Figure 8 shows the control surface of $x_{c}$ and $y_{c}$ for the non-fuzzy case (4). The control surfaces of the fuzzy approximations (7) for the $30^{{}^{\circ}}$ and $7.5^{{}^{\circ}}$ sectors are shown in Figure 9 and Figure 10. The resolution $30^{{}^{\circ}}$ (Figure 9) shows a very high deviation compared to the non-fuzzy approach (Figure 8), which decreases further down to the resolution $7.5^{{}^{\circ}}$ (Figure 10). This explains the high differences between the measured and computed standard deviations and correlation coefficients, in particular for sector sizes of $30^{{}^{\circ}}$ and higher.

### 6.2. Sigma-Point Method

**Two-inputs–two-outputs**:

The simulation of the sigma-point method is based on a Matlab implementation of an unscented Kalman filter by [25]. The first example deals with the 2-inputs–2-outputs case in which only the orientations are taken into account, but the disturbances of the positions of the robot and human are not part of the sigma-point calculation. A comparison between the computed and measured covariance shows a very good match. The same holds for the standard deviations ${\sigma_{x}}_{c},{\sigma_{y}}_{c}$. A comparison with the statistical linearization shows a good match as well (see Table 2, rows 1 and 2).

**Figure 11:**
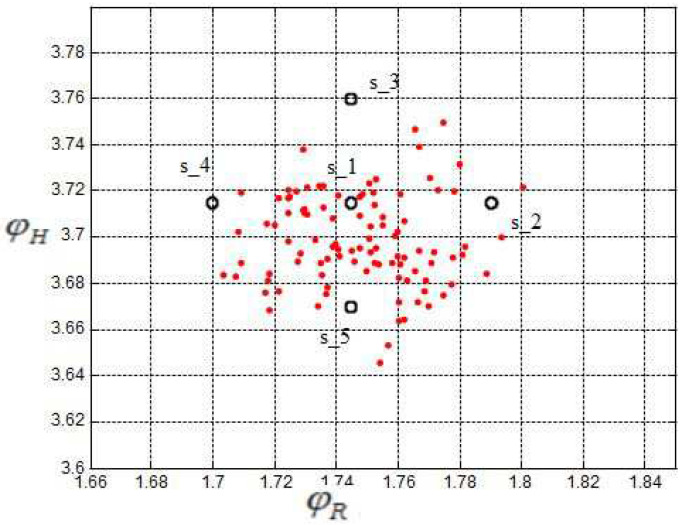
Sigmapoints, input, units of $\phi_{R}$ and $\phi_{H}$ in rad.

**Figure 12:**
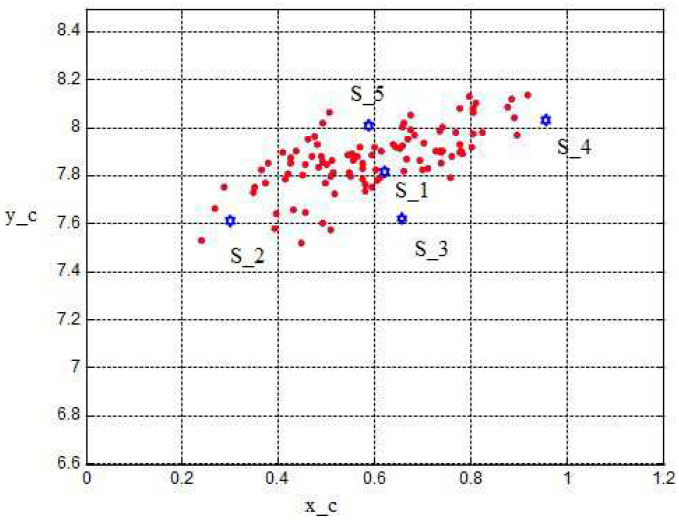
Sigmapoints, output.

A view at the sigma points presents the following results: Figure 11 shows the two-dimensional distribution of the orientation angles $(\phi_{R}$, $\phi_{H})$ and the corresponding sigma points $s_{1},\ldots ,s_{5}$ where $s_{1}$ denotes the mean value. Figure 12 shows the two-dimensional distribution of the intersection coordinates $\left(x_{c},y_{c}\right)$ with the sigma points $S_{1},\ldots ,S_{5}$. $S_{1}$ denotes the mean value and $S_{1},\ldots ,S_{5}$ are distributed in such a way that the $s_{i}$ are transformed into $S_{i}$, i=1…5. From both figures, an optimal selection of both $s_{1},\ldots ,s_{5}$ and $S_{1},\ldots ,S_{5}$ can be observed, which results in a good match of the computed and measured standard deviations $\sigma_{x_{c}}$.

**Six-inputs–two-outputs**:

The 6-inputs–2-outputs example shows that the additional consideration of 4 input position coordinates with $\sigma_{x_{R}}=0.02$ leads to similar results both for the computed and measured covariances and between the sigma-point method and statistical linearization (see $P\left(7,7\right)={\sigma_{x}}_{c}^{2}$, $P\left(8,8\right)={\sigma_{y}}_{c}^{2}$ and $covar\left(7,7\right)={\sigma_{x}}_{m}^{2}$, $covar\left(8,8\right)={\sigma_{y}}_{m}^{2}$, and ${\sigma_{x}}_{c}^{2}$—computed, and ${\sigma_{x}}_{m}^{2}$—the measured variation). Table 2 shows the covariance submatrix considering the output positions only.

Computed covariance:

(60) $$\begin{array}{ccc} P & = & 10^{-1}\times \;\left(\begin{array}{cccccccc} 0.004 & -0.000 & -0.000 & 0.000 & -0.000 & -0.000 & -0.030 & -0.018\\ -0.000 & 0.004 & 0.000 & -0.000 & -0.000 & -0.000 & 0.003 & -0.017\\ -0.000 & 0.000 & 0.004 & 0.000 & -0.000 & -0.000 & 0.004 & 0.002\\ 0.000 & -0.000 & 0.000 & 0.004 & -0.000 & -0.000 & 0.001 & 0.000\\ -0.000 & -0.000 & -0.000 & -0.000 & 0.004 & 0.000 & 0.000 & -0.002\\ -0.000 & -0.000 & -0.000 & -0.000 & 0.000 & 0.004 & -0.001 & 0.004\\ -0.030 & 0.003 & 0.004 & 0.001 & 0.000 & -0.001 & 0.235 & 0.127\\ -0.018 & -0.017 & 0.002 & 0.000 & -0.002 & 0.004 & 0.127 & 0.165 \end{array}\right)\\ {\sigma_{x}}_{c} & = & 0.153,\;{\sigma_{y}}_{c}=0.122 \end{array}$$

Measured covariance:

(61) $$\begin{array}{ccc} covar & = & 10^{-1}\times \;\left(\begin{array}{cccccccc} 0.004 & 0.000 & 0.000 & 0.000 & 0.000 & -0.000 & -0.028 & -0.020\\ 0.000 & 0.004 & 0.000 & 0.001 & 0.000 & -0.000 & 0.000 & -0.020\\ 0.000 & 0.000 & 0.004 & -0.000 & 0.001 & -0.001 & 0.003 & 0.001\\ 0.000 & 0.001 & -0.000 & 0.004 & -0.000 & -0.000 & -0.000 & -0.003\\ 0.000 & 0.000 & 0.001 & -0.000 & 0.005 & -0.000 & -0.001 & -0.006\\ -0.000 & -0.000 & -0.001 & -0.000 & -0.000 & 0.005 & -0.000 & 0.005\\ -0.028 & 0.000 & 0.003 & -0.000 & -0.001 & -0.000 & 0.213 & 0.131\\ -0.020 & -0.020 & 0.001 & -0.003 & -0.006 & 0.005 & 0.131 & 0.182 \end{array}\right)\\ {\sigma_{x}}_{c} & = & 0.145,\;{\sigma_{y}}_{c}=0.134 \end{array}$$

**Two-inputs–two-outputs, direct and inverse solution**

The next example shows the computation of the direct and inverse cases. In the direct case, we obtain again similar values between the computed and measured covariances and, with this, the standard deviations. The results of the inverse solution lead to similar values of the original inputs (orientations $x_{1}=\phi_{R},x_{2}=\phi_{H}$) (see Table 2). The simulations of the fuzzy versions showed the same similarities and can therefore be left out here.

**Two-inputs–two-outputs, moving robot–human**

**Figure 13:**
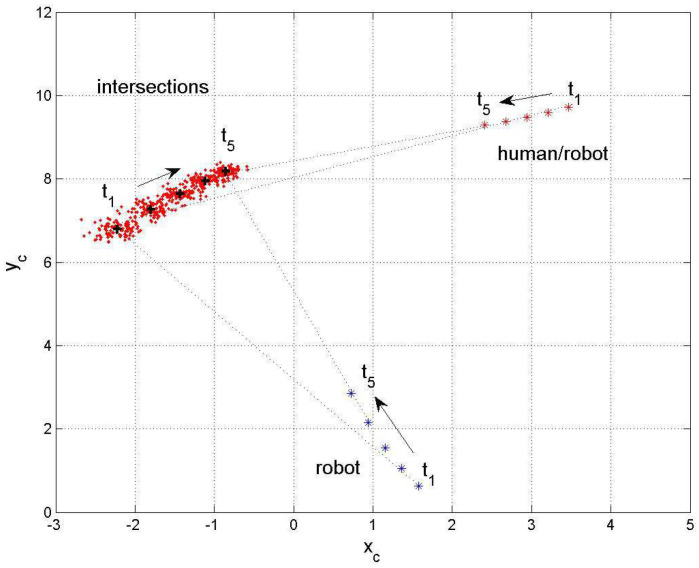
Moving robot and human.

The next example deals with the robot and human in motion. Figure 13 shows the positions and orientations of the robot and human at selected time steps $t_{1}\ldots t_{5}$ and the development of the corresponding intersections $x_{c}$.

**Figure 14:**
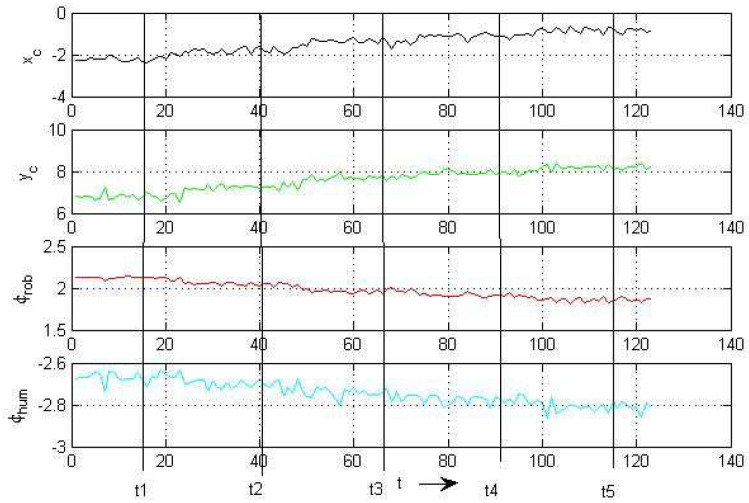
Time plot, robot and human.

Figure 14 shows the corresponding time plot. The time steps $t_{1}\ldots t_{5}$ are taken at 0.58 s, …,4.58 s with a time distance of 1s, which is 25 time steps of 0.04 s each. The robot and human start at

$x_{R}={\left(x_{R},y_{R}\right)}^{T}={\left(2,0\right)}^{T}$m
$x_{H}={\left(x_{H},y_{H}\right)}^{T}={\left(4,10\right)}^{T}$m
with the velocities
${\dot{x}}_{R}\left(k\right)=-0.21$ m/s;
${\dot{y}}_{R}\left(1\right)=+0.24$ m/s;
${\dot{x}}_{H}\left(k\right)=-0.26$ m/s;
${\dot{y}}_{H}\left(1\right)=-0.24$ m/s.
*k* is the time step.

The *x* components of the velocities ${\dot{x}}_{R}\left(k\right)$ and ${\dot{x}}_{H}\left(k\right)$ stay constant during the whole simulation.

The *y* components change their velocities with constant factors

$$\begin{array}{ccc} {\dot{y}}_{R}\left(k+1\right) & = & K_{R}\cdot {\dot{y}}_{R}\left(k\right)\\ {\dot{y}}_{H}\left(k+1\right) & = & K_{H}\cdot {\dot{y}}_{H}\left(k\right) \end{array}$$

where $K_{R}=1.2$ and $K_{H}=0.9$. The orientation angles start with the following:

$\phi_{R}=1.78$ rad;
$\phi_{H}=3.69$ rad.

They change their values every second according to the direction of motion.

From both plots, one observes an expected decrease in the output standard deviations for a mutual decrease in their distances to the specific intersection and a good match between the computed and measured values $x_{c}$ (see Table 3). With the information about the distance of the robot and the standard deviation from and at the expected intersection, respectively, it becomes possible to plan either an avoidance strategy or mutual cooperation between the robot and human.

## 7. Summary and Conclusions

The content of this work is the prediction of encounter situations of mobile robots and human agents in shared areas by analyzing planned/intended trajectories in the presence of uncertainties and system and observation noise. In this context, the problem of intersections of trajectories with respect to system uncertainties and Gaussian noise of the position and orientation of the agents involved is discussed. The problem is addressed by two methods: the statistical linearization of distributions and the sigma-point transformation of the distribution parameters. The positions and orientations of the robot and human are corrupted with Gaussian noise represented by the parameters’ mean and standard deviation. The goal is to calculate the mean and standard deviation/variation at the intersection via the nonlinear relation between the positions/orientations of the robot and human, on the one hand, and the position of the intersection of their intended trajectories, on the other hand.

This analysis is realized by the statistical linearization of the nonlinear relation between the statistics of the robot and human (input) and the statistics of the intersection (output). The output results are the mean and standard deviation of the intersection as functions of the input parameters’ mean and standard deviation of the positions and orientations of robot and human. This work is first carried out for two-input–two-output relations (two orientations of the robot–human and two intersection coordinates) and then for six inputs–two outputs (two orientations and four position coordinates of the robot–human and two intersection coordinates). These cases were extended to their fuzzy versions by different Takagi–Sugeno (TS) fuzzy approximations and compared with the non-fuzzy case. Up to a certain resolution, the approximation works as accurately as the original non-fuzzy version. For the two-input–two-output case, an inverse solution is derived, except for the six-input–two-output case because of the undetermined nature of the differential input–output relation.

The sigma-point transformation aims at transforming/propagating distribution parameters—the sigma points—directly through nonlinearities. The transformed sigma points are then converted into the distribution parameters’ mean and covariance matrix. The sigma-point transformation is closely connected to the unscented Kalman filter, which is used in the example of the robot and human in motion. The specialty of the example is a computed virtual system output (“observation”)—the intersection of two intended trajectories—where the corresponding output uncertainty is a sum of the transformed position/orientation noise and the computational uncertainty from the fuzzy approximation. In total, the comparison between the computed and measured covariances shows a very good match and the comparison with the statistical linearization shows good coincidences as well. Both the sigma-point transformation and the differential statistical linearization scales for more than two variables linearly. Their computational complexity is in the same order [13]. However, if the model is nonlinear, then the differential linearization (EKF) serves as the first-order or second-order approximating estimator. If the system is highly nonlinear, the EKF may diverge and the sigma-point approach produces typically better results. In summary, a prediction of the accuracy of human–robot trajectories using the methods presented in this work increases the performance of human–robot collaboration and human safety. In future work, this method can be used for robot–human scenarios in factory workshops and for robots working in complicated environments like rescue operations in cooperation with human operators.

## Figures and Tables

**Table 1:** Standard deviations and fuzzy and non-fuzzy results.

Input Std	0.02 Gauss, Bell Shaped (GB)				Gauss	0.05 GB
sector size/°	$60^{{}^{\circ}}$	$30^{{}^{\circ}}$	$15^{{}^{\circ}}$	$7.5^{{}^{\circ}}$	$7.5^{{}^{\circ}}$	$7.5^{{}^{\circ}}$
non-fuzz ${\sigma_{x}}_{c}$	0.143	0.140	0.138	**0.125**	**0.144**	**0.366**
fuzz ${\sigma_{x}}_{c}$	0.220	0.184	0.140	**0.126**	**0.144**	**0.367**
non-fuzz ${\sigma_{x}}_{m}$	0.160	0.144	0.138	**0.126**	**0.142**	**0.368**
fuzz ${\sigma_{x}}_{m}$	0.555	0.224	0.061	**0.225**	**0.164**	**0.381**
non-fuzz ${\sigma_{y}}_{c}$	0.128	0.132	0.123	**0.114**	**0.124**	**0.303**
fuzz ${\sigma_{y}}_{c}$	0.092	0.087	0.120	**0.112**	**0.122**	**0.299**
non-fuzz ${\sigma_{y}}_{m}$	0.134	0.120	0.123	**0.113**	**0.129**	**0.310**
fuzz ${\sigma_{y}}_{m}$	0.599	0.171	0.034	**0.154**	**0.139**	**0.325**
non-fuzz ${\rho_{xy}}_{c}$	0.576	0.541	0.588	**0.561**	**0.623**	**0.669**
fuzz ${\rho_{xy}}_{c}$	−0.263	0.272	0.478	**0.506**	**0.592**	**0.592**
non-fuzz ${\rho_{xy}}_{m}$	0.572	0.459	0.586	**0.549**	**0.660**	**0.667**
fuzz ${\rho_{xy}}_{m}$	0.380	0.575	0.990	**0.711**	**0.635**	**0.592**

**Table 2:** Covariances, standard deviations—computed and measured.

Outputs	Covariance, Computed	Covariance, Measured	${\sigma_{x}}_{c}$, Comp/Meas	${\sigma_{y}}_{c}$, Comp/Meas
2 inputs	$P=\;\left(\begin{array}{cc} 0.0213 & 0.0114\\ 0.0114 & 0.0159 \end{array}\right)$	$covar=\;\left(\begin{array}{cc} 0.0264 & 0.0146\\ 0.0146 & 0.0166 \end{array}\right)$	0.145/0.144	0.126/0.134
2 inputs, stat. lin.	-	-	0.144/0.142	0.124/0.129
6 inputs	$P=\;\left(\begin{array}{cc} 0.0235 & 0.0127\\ 0.0127 & 0.0165 \end{array}\right)$	$covar=\;\left(\begin{array}{cc} 0.0213 & 0.0131\\ 0.0131 & 0.0182 \end{array}\right)$	0.135/0.145	0.122/0.134
Direct solution	$P=\;\left(\begin{array}{cc} 0.0234 & 0.0133\\ 0.0133 & 0.0151 \end{array}\right)$	$covar=\;\left(\begin{array}{cc} 0.0264 & 0.0146\\ 0.0146 & 0.0166 \end{array}\right)$	0.152/0.162	0.128/128
Inverse solution	$P=10^{-3}\times \;\left(\begin{array}{cc} 0.4666 & 0.0522\\ 0.0522 & 0.4744 \end{array}\right)$	$covar=10^{-3}\times \;\left(\begin{array}{cc} 0.4841 & -0.0190\\ -0.0190 & 0.396 \end{array}\right)$	0.0215/0.0220	0.0217/0.0190

**Table 3:** Covariances, standard deviations—computed and measured, moving robot–human.

Outputs	Covariance, Computed	Covariance, Measured	${\sigma_{x}}_{c}$, Comp/Meas	${\sigma_{y}}_{c}$, Comp/Meas
$t_{1}$	$P=\;\left(\begin{array}{cc} 0.0220 & 0.0017\\ 0.0017 & 0.0163 \end{array}\right)$	$covar=\;\left(\begin{array}{cc} 0.0246 & -0.0002\\ -0.0002 & 0.0202 \end{array}\right)$	0.148/0.156	0.127/0.142
$t_{2}$	$P=\;\left(\begin{array}{cc} 0.0198 & 0.0023\\ 0.0023 & 0.0138 \end{array}\right)$	$covar=\;\left(\begin{array}{cc} 0.0222 & 0.0018\\ 0.0018 & 0.0153 \end{array}\right)$	0.140/0.148	0.117/0.123
$t_{3}$	$P=\;\left(\begin{array}{cc} 0.0168 & 0.0030\\ 0.0030 & 0.0107 \end{array}\right)$	$covar=\;\left(\begin{array}{cc} 0.0140 & 0.0040\\ 0.0040 & 0.0088 \end{array}\right)$	0.129/0.118	0.103/0.093
$t_{4}$	$P=\;\left(\begin{array}{cc} 0.0151 & 0.0029\\ 0.0029 & 0.0083 \end{array}\right)$	$covar=\;\left(\begin{array}{cc} 0.0127 & 0.0014\\ 0.0014 & 0.0073 \end{array}\right)$	0.122/0.112	0.091/0.085
$t_{5}$	$P=\;\left(\begin{array}{cc} 0.0125 & 0.0023\\ 0.0023 & 0.0061 \end{array}\right)$	$covar=\;\left(\begin{array}{cc} 0.0102 & 0.0030\\ 0.0030 & 0.0056 \end{array}\right)$	0.112/0.101	0.078/0.074

## Data Availability

Data are contained within the article.
